# Genetic polymorphism of the ovine *MAP3K5* gene and its association with body size traits in Hu sheep of China

**DOI:** 10.5194/aab-66-71-2023

**Published:** 2023-02-16

**Authors:** Xiaobin Yang, Weimin Wang, Deyin Zhang, Xiaolong Li, Yukun Zhang, Yuan Zhao, Liming Zhao, Jianghui Wang, Dan Xu, Jiangbo Cheng, Wenxin Li, Bubo Zhou, Changchun Lin, Xiwen Zeng, Rui Zhai, Zongwu Ma, Jia Liu, Panpan Cui, Xiaoxue Zhang

**Affiliations:** 1 College of Animal Science and Technology, Gansu Agricultural University, Lanzhou, Gansu 730070, China; 2 The State Key Laboratory of Grassland Agro-ecosystems, College of Pastoral Agriculture Science and Technology, Lanzhou University, Lanzhou, Gansu 730020, China

## Abstract

As an excellent local sheep breed in China, Hu sheep have the characteristics of producing more lambs and good motherhood. The purpose of this study was to identify the polymorphism of the mitogen-activated protein kinase 5 (*MAP3K5*) gene and determine whether it was associated with the body size traits (body height, body length, chest circumference, and cannon circumference) in Hu sheep. The polymorphism of *MAP3K5* was identified by using PCR amplification, followed by Sanger sequencing, and KASPar (Kompetitive Allele Specific PCR) technology was used for genotyping subsequently. The results of the association analysis showed that *MAP3K5* (g.205261 A > G) was significantly associated with body height at 80, 100, 140, 160, and 180 d; body length at 80 and 160 d; chest circumference at 100, 140, 160, and 180 d; and cannon circumference at 100 and 120 d, respectively. The results of qRT-PCR showed that the expression level of *MAP3K5* in the heart was significantly higher (P < 0.05) than in the other 10 tissues. In summary, the *MAP3K5* mutation loci may be used as a candidate molecular marker for the body size traits of Hu sheep.

## 1 Introduction

As early domesticated animals, sheep provide meat, milk, and other products for humans (Awawdeh, 2011; Li et al., 2018). Hu sheep originated in the Taihu region and is one of the local breeds in China. It has the characteristics of high reproduction rate, fast growth, and more robust environmental adaptability (H et al., 2020; Chen et al., 2020). With the development of the economy and improvement in living standards, Chinese demand for sheep is increasing year after year. Of course, the quality of the sheep is a very important issue, but we should also pay attention to the production of sheep. Breeding will play a vital role in resolving the issue of insufficient supply of sheep (H et al., 2020). Excellent livestock breeds play an essential role in livestock development (Abraham et al., 2018; Quan et al., 2020). Body size traits include body height, body length, chest circumference, and cannon circumference, etc., which are influenced by many factors and also play significant roles in production practice. There are also various methods of animal breeding; compared to traditional breeding methods, researchers indicate that molecular marker technology will help establish excellent traits. Pedersen et al. (2009) showed that marker-assisted selection is a useful selection strategy when comparing BLUP (best linear unbiased prediction), QTL (quantitative trait locus), and MAS (molecular marker-assisted selection) (Pedersen et al., 2009). Therefore, in the breeding practice, we can use molecular marker technology to select sheep with more prominent body size traits for production, to maximize the expansion of production efficiency, reduce production costs, and increase the supply of meat products.

Mitogen-activated protein kinase 5 (*MAP3K5*), also known as apoptosis signal-regulating kinase 1 (*ASK1*), is a member of the mitogen-activated protein kinase family (MAPK) (Pu et al., 2016; Takenaka et al., 2020). The MAPK family was first elucidated in 1994 and is involved in the control of the cell cycle, apoptosis, and differentiation. Kyriakis and Avruch (2012) demonstrated that the MAPK signaling pathway plays an important role in the cellular physiology of mammals (Kyriakis and Avruch, 2012). *MAP3K5* plays an important role in cell response cascades caused by environmental changes. A study by Sirotkin et al. (2008) found that the transcription factor p53, which is mainly located in the nucleus, can stimulate the expression of *MAP3K5*, which is mainly located in the cytoplasm (Sirotkin et al., 2008). In addition, the study of mice, Hattori et al. (2009) found that apoptosis induced by tumor necrosis factor α or hydrogen peroxide was inhibited in mouse embryonic fibroblasts deficient in *ASK1* (Hattori et al., 2009). Harada et al. (2006) found that inhibition of the ASK1-p38 pathway could be used to treat neurodegenerative diseases including glaucoma (Harada et al., 2006). Some researchers have shown that *MAP3K5* was highly expressed in the heart of Duroc pigs, which indicated that this was related to their high metabolic capacity (Pu et al., 2016). Although there are many studies related to this gene and previous studies have shown that the *MAP3K5* gene can affect physiological processes in animal cells, the influence of the *MAP3K5* gene on sheep body size traits is not clear. Consequently, we explored the relationship between the single nucleotide polymorphism (SNP) of the *MAP3K5* gene and the body size traits of Hu sheep in this study, which has a certain value for sheep breeding and screening of sheep with excellent body size traits.

## 2 Materials and methods

### 2.1 Experimental animals and DNA extraction

The animals used in this study were farmed in Minqin Defu Agriculture Co. Ltd. (Gansu, China). All lambs were purchased from four commercial sheep farms (including Jinchang Zhongtian Sheep Industry Co. Ltd., Gansu Sanyangjinyuan Husbandry Co. Ltd., Gansu Zhongsheng Huamei Sheep Industry Development Co. Ltd., and Wuwei Pukang Breeding Co. Ltd. in Gansu Province, China). All these sheep received a standardized vaccination program before weaning.

All the lambs had a 10 d experimental preparation period, a 14 d adaptation period to the new conditions of the environment, and a 100 d experimental period. The feeding, management conditions and environment of the whole experiment were consistent; furthermore, ad libitum feeding was followed. A total of 1388 male Hu sheep were housed in a single column of 0.8 × 1 m from 56 to 180 d old. Body size traits of all lambs were recorded, which included body height, body length, chest circumference, and cannon circumference at the 80th, 100th, 120th, 140th, 160th, and 180th days before feeding in the morning. During the whole experiment process, it was ensured that one or two people in charge measured the sheep's body size trait data as much as possible, to reduce unnecessary measurement errors. From the jugular vein of all sheep, 5 mL of blood was collected when they were at 180 d, and DNA was extracted using a kit (TransGen Biotech, Beijing, China), which was dissolved in an elution buffer. And then they were dissolved in an elution buffer (10 mM tris hydrochloride and 1 mM ethylenediamine tetra acetic acid, pH 8.0). NANODROP ONE (Thermo Fisher Scientific, USA) was used to detect the concentration and purity of the obtained DNA templates, which were temporarily stored in -20 ${}^{\circ }$C until needed.

### 2.2 SNP identification and genotyping

The specific PCR primers for *MAP3K5* (GenBank accession NC_040259.1) were designed by Oligo 7 and Primer 5 software. The most suitable annealing temperature for the primer of the *MAP3K5* gene was determined by a gradient PCR instrument. Then the PCR products were produced from amplification with mixed DNA as the template, which was sequenced to identify the SNP in *MAP3K5*. The specific PCR primers for *MAP3K5 *are shown in Table 1. The PCR product was obtained by using the mixed DNA of 10 sheep randomly selected from the experimental population as a template (Fig. 2). The PCR was performed using a mixture of a total volume of 35 µL, containing 17.5 µL Master Mix (TSINGKE Biological Technology, Beijing, China), 1.0 µL forward and reverse primers each, 1.5 µL template DNA, and 14 µL of H${}_{2}$O. Before that, the optimum temperature screening of the *MAP3K5* gene was conducted, and the results are shown in Figs. S1a and b in the Supplement. The cycling conditions were as follows: 3 min at 94 ${}^{\circ }$C, followed by 30 s at 94 ${}^{\circ }$C, 30 s at 45–65 ${}^{\circ }$C, 30 s at 72 ${}^{\circ }$C for 35 cycles, and finally 5 min at 72 ${}^{\circ }$C. As reported by a previous study, the SNP identified *MAP3K5*, and genotype-specific primers were used for competitive PCR (KASPar) (He et al., 2014). The information on the primers used in KASPar is showed in Table 1. In this study, a total of 1355 sheep were successfully genotyped.

**Table 1:** Primer sequences and PCR conditions and primers used to the KASPar technology.

Gene	Primer	Primer sequence (5${}^{\prime }$–3${}^{\prime }$)	Size (bp)	$T_{m}$ (${}^{\circ }$C)	
*MAP3K5*	*MAP3K5*-SNP-F	GACAGACAGCCATCTACACT	373	53	
	*MAP3K5*-SNP-R	ACACAATGAAAATCGTGCAG			
Gene	Primer	Primer sequence (5${}^{\prime }$–3${}^{\prime }$)			
*MAP3K5*	Primer_AlleleX	GAAGGTGACCAAGTTCATGCTCGTCTTTTCTTAAAGACCCAGGTCA			
	Primer_AlleleY	GAAGGTCGGAGTCAACGGATTGTCTTTTCTTAAAGACCCAGGTCG			
	Primer_Common	GAGGTTTATTCAAAATTTGGTTTAACTCCAC			

### 2.3 Tissue expression analysis

Six sheep's heart, liver, spleen, lung, kidney, rumen, lymph, muscle, duodenum, tail fat, and bone tissues were randomly selected, and RNA was extracted from these tissues with Transzol up (TransGen, Beijing, China) and chloroform. A reverse transcriptase kit (Accurate Biotechnology Co., Ltd, Hunan, China) was used to reverse transcribe the RNA into cDNA and then qRT-PCR after the detection concentration and quality are qualified. Each sample will undergo four technical replicates and six biological replicates. The qRT-PCR components were as follows: 6.4 µL RNase-free water, 10 µL 2× SYBR Green PCR Master Mixture (Takara Biotechnology), 0.8 µL upstream and downstream primers, and 2.0 µL template cDNA. The apparatus of LightCycler^®^ 480 (Roche, Basel, Sweden) was used for experimental analysis, and the reaction conditions of qRT-PCR were as follows: 94 ${}^{\circ }$C for 5 min, followed by 34 cycles of 94 ${}^{\circ }$C for 30 s, 57 ${}^{\circ }$C for 30 s, 72 ${}^{\circ }$C for 30 s, and finally 72 ${}^{\circ }$C for 5 min (Wang et al., 2014). The data were analyzed with the 2${}^{-\mathrm{CT}}$ method (Livak and Schmittgen, 2001).

### 2.4 Statistical analysis

The genotype frequency, allele frequency, expected heterozygosity ($H_{e}$), expected homozygosity ($H_{o}$), allele number ($N_{e}$), polymorphism information content (PIC), and the P value of Hardy–Weinberg equilibrium ($P_{\mathrm{HWE}}$) were calculated based on previous research (Zhao et al., 2013). SPSS 24.0 software was used to analyze the relationship between different genotypes and body size traits. Body size traits based on the following general linear model, where genotypes, seasons, and batches were fixed factors (main effects), and the phenotypic data of experimental populations corresponding to different genotypes was the dependent variable. The specific model was defined as follows:

$$Y_{ijk}=\mu +{\mathrm{genotype}}_{i}+{\mathrm{batch}}_{j}+{\mathrm{season}}_{k}+\varepsilon_{ijk}.$$

In this model, $Y_{ijk}$ represents the phenotype of body size traits and μ is the average. Genotype${}_{i}$ represents the effect of ith genotypes. Batch${}_{j}$ represents the effect of jth batch (j=2, 3, 4, 5, 6, 7). Season${}_{k}$ represents the effect of kth season (k=1, 2) and $\varepsilon_{ijk}$ represents random error. Duncan's test was used to analyze the association between different genotypes and phenotypic traits. P<0.05 indicated statistical significance.

## 3 Results

### 3.1 Descriptive statistics of body size traits

The results of descriptive statistics of body size traits in different stages are shown in Table 2. With increasing age, body size traits increased to varying degrees. The correlation analysis of body size traits is shown in Fig. 1, which suggests that the traits of body height and body length have the highest correlation at the same stage. In contrast, the correlation between body height and cannon circumference was relatively low. It was worth noting that these four traits all showed a positive correlation.

**Figure 1:**
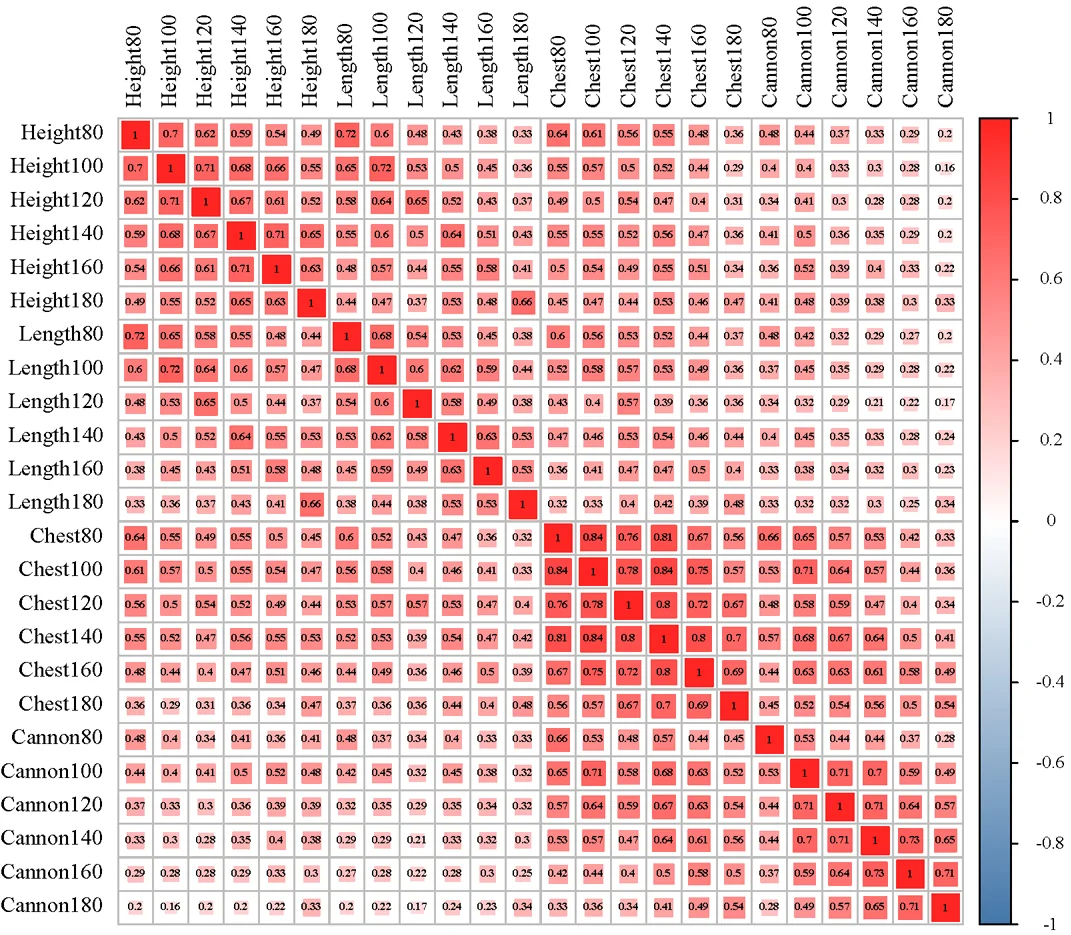
Correlation analysis of body height (height), body length (length), chest circumference (chest), and cannon circumference (cannon) of sheep.

**Table 2:** Descriptive statistics of body size traits.

Items	Day	Mean	SD	Min	Max	CV (%)
Body height	80	54.56	3.36	43.00	69.00	6.15
(cm)	100	58.04	3.53	46.50	70.00	6.08
	120	61.26	3.35	48.00	72.00	5.48
	140	63.83	3.48	51.00	75.00	5.45
	160	65.97	3.56	51.50	79.00	5.40
	180	68.29	3.73	53.00	85.00	5.46
Body length	80	56.14	3.50	46.00	69.50	6.23
(cm)	100	61.10	3.92	48.00	77.00	6.42
	120	65.27	3.92	53.00	83.00	6.00
	140	67.87	3.69	56.00	83.00	5.44
	160	70.38	3.58	58.00	86.00	5.09
	180	73.26	4.37	61.00	87.00	5.97
Chest circumference	80	61.17	4.92	47.00	75.00	8.05
(cm)	100	65.10	5.12	47.00	85.00	7.86
	120	69.61	4.58	55.00	87.00	6.58
	140	73.39	4.89	58.00	89.00	6.67
	160	77.59	4.98	62.50	99.00	6.41
	180	82.45	4.94	64.00	100.00	6.00
Cannon	80	6.79	0.64	5.00	9.00	9.43
circumference	100	7.16	0.68	5.00	9.00	9.44
(cm)	120	7.44	0.64	5.50	9.50	8.62
	140	7.72	0.63	6.00	10.00	8.14
	160	7.90	0.65	6.20	10.00	8.21
	180	8.12	0.63	6.50	10.00	7.73

### 3.2 SNP of sheep *MAP3K5*

A silent mutation at g.205261 A > G was identified by sequencing in *MAP3K5* (Fig. 3). The SNP g.205261 A > G in *MAP3K5* was located in the intron 25. This SNP locus was typed by KASPar technology, three genotypes were identified: GG, GA, and AA (Fig. 4).

**Figure 2:**
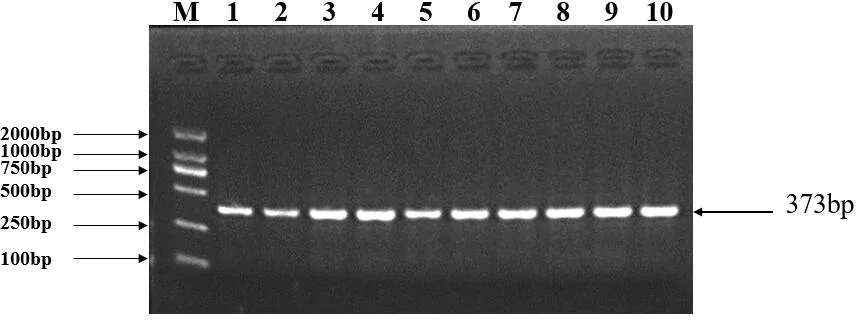
PCR amplification of *MAP3K5*. Lanes 1–10: target fragments; M: DL2000 DNA marker.

**Figure 3:**
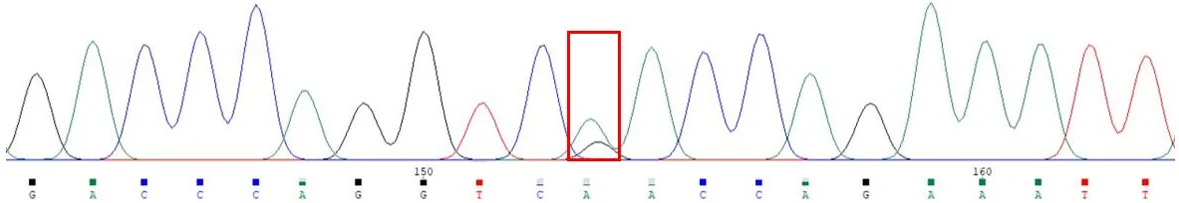
Sequencing peak map of *MAP3K5* gene.

**Figure 4:**
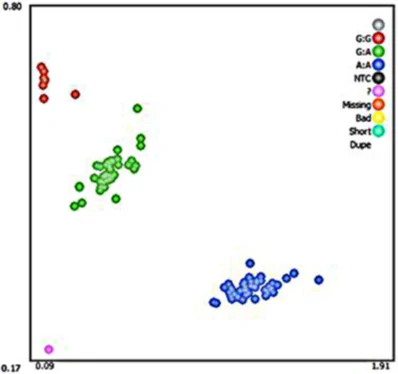
Genotyping map of *MAP3K5* g.205261 A > G. Red, green, and blue specifically refer to three genotypes: GG, GA, and AA genotypes. KASPar genotyping failure is represented by pink.

### 3.3 Genetic parameters of SNP

The genetic parameters of the SNP g.205261 A > G are shown in Table 3. The genotypic frequencies of AA, GA, and GG were 0.406, 0.469, and 0.125, respectively. In addition, the results showed that A was A-dominant allele, and its allele frequency was 0.640. In addition, the $H_{e}$, $H_{o}$, $N_{e}$, PIC, and $P_{\mathrm{HWE}}$ for *MAP3K5* were 0.461, 0.539, 1.855, 0.433, and 0.722, respectively.

**Table 3:** Genetic parameters of SNP at the *MAP3K5* loci.

Loci	No.	Genotype	Genotype	Allele	Allele	$H_{e}$	$H_{o}$	$N_{e}$	PIC	$P_{\mathrm{HWE}}$
			frequency		frequency					
*MAP3K5* g.205261 A > G	557	AA	0.411	A	0.643	0.459	0.541	1.848	0.354	0.722
	629	GA	0.464							
	169	GG	0.125	G	0.357					

Note: expected heterozygosity ($H_{e}$), expected homozygosity ($H_{o}$), allele number ($N_{e}$), polymorphism information content (PIC), and P value of Hardy–Weinberg equilibrium ($P_{\mathrm{HWE}}$).

### 3.4 Association analysis of body size traits at different stages

A general linear statistical model was used to estimate the association between the SNP g.205261 A > G of the *MAP3K5* gene and body size traits. The results showed that the *MAP3K5* gene had a certain association with different body size traits at different stages, which were shown in Table 4. Among the body height, there was a significant association on the 80th, 100th, 140th, 160th, and 180th days (P<0.05). For the body length, there was a significant association between the 80th and 160th days (P<0.05). For the chest circumference, there was a significant association on the 100th, 140th 160th, and 180th days (P<0.05). Among the cannon circumference, there was a significant association on the 100th and 120th days (P<0.05). The results showed that the body height, body length, chest circumference, and cannon circumference of AA genotype sheep were significantly higher than those of GG genotype sheep, which proved that AA genotype was the main effect on sheep body height, body length, chest circumference, and cannon circumference.

**Table 4:** Association of genotypes of *MAP3K5* (g.205261 A > G) with body size traits.

Items	Day	AA	GA	GG
No.		557	629	169
Body height	80	54.74 ± 3.44${}^{a}$	54.54 ± 3.32${}^{a}$	54.03 ± 3.18${}^{b}$
(cm)	100	58.23 ± 3.58${}^{a}$	58.00 ± 3.56${}^{\mathrm{ab}}$	57.70 ± 3.23${}^{b}$
	120	61.32 ± 3.36	61.22 ± 3.37	61.21 ± 3.30
	140	63.99 ± 3.34${}^{a}$	63.83 ± 3.59${}^{a}$	63.28 ± 3.48${}^{b}$
	160	66.16 ± 3.57${}^{a}$	65.95 ± 3.61${}^{a}$	65.44 ± 3.33${}^{b}$
	180	68.44 ± 3.64${}^{a}$	68.27 ± 3.78${}^{\mathrm{ab}}$	67.85 ± 3.81${}^{b}$
Body length	80	56.39 ± 3.63${}^{a}$	56.06 ± 3.37${}^{\mathrm{ab}}$	55.64 ± 3.49${}^{b}$
(cm)	100	61.23 ± 4.09	61.07 ± 3.81	60.77 ± 3.81
	120	65.41 ± 3.85	65.19 ± 4.01	65.09 ± 3.84
	140	68.00 ± 3.65	67.85 ± 3.76	67.50 ± 3.59
	160	70.70 ± 3.74${}^{a}$	70.25 ± 3.49${}^{\mathrm{ab}}$	69.79 ± 3.31${}^{b}$
	180	73.48 ± 4.30	73.16 ± 4.43	72.92 ± 4.41
Chest	80	61.33 ± 5.06	61.09 ± 4.89	60.91 ± 4.57
circumference	100	65.27 ± 5.18${}^{a}$	65.10 ± 5.12${}^{\mathrm{ab}}$	64.53 ± 4.91${}^{b}$
(cm)	120	69.67 ± 4.43	69.66 ± 4.71	69.21 ± 4.59
	140	73.67 ± 5.01${}^{a}$	73.33 ± 4.89${}^{a}$	72.67 ± 4.45${}^{b}$
	160	77.76 ± 4.94${}^{a}$	77.66 ± 5.07${}^{a}$	76.78 ± 4.69${}^{b}$
	180	82.70 ± 4.98${}^{a}$	82.51 ± 5.01${}^{a}$	81.42 ± 4.43${}^{b}$
Cannon	80	6.83 ± 0.65	6.76 ± 0.64	6.76 ± 0.60
circumference	100	7.17 ± 0.68${}^{a}$	7.18 ± 0.67${}^{a}$	7.04 ± 0.68${}^{b}$
(cm)	120	7.48 ± 0.63${}^{a}$	7.43 ± 0.65${}^{a}$	7.34 ± 0.63${}^{b}$
	140	7.73 ± 0.62	7.72 ± 0.63	7.67 ± 0.63
	160	7.92 ± 0.63	7.90 ± 0.66	7.86 ± 0.66
	180	8.11 ± 0.62	8.13 ± 0.64	8.10 ± 0.63

Note: all values are expressed as mean ± standard deviation. Mean ± SD showing different superscripts are significantly different at P<0.05.

### 3.5 Analysis of RNA expression profiles in various tissues of sheep

The mRNA sequences of sheep *MAP3K5* g.205261 G > A (GenBank accession no. XM_042253518) were used as templates (Table 5). The RNA expression profiles showed that the *MAP3K5* gene was widely expressed in these tissues, and the expression level in the heart was significantly higher than that of the other tissues (including lung, kidney, rumen, tail fat, liver, spleen, lymph, muscle, duodenum, and bone; P<0.05, Fig. 5). The expression levels of the *MAP3K5* gene in different genotypes of Hu sheep are shown in Fig. 6. The results showed that there was no significant difference in the expression levels of the *MAP3K5* gene in the heart and muscle tissues of Hu sheep with AA and GG genotypes, while the expression levels of the *MAP3K5* gene in the liver and lymph tissues of Hu sheep with an AA genotype were significantly higher than those of Hu sheep with a GG genotype (P<0.05).

**Table 5:** Primers used for qRT-PCR for the ovine *MAP3K5* gene.

Gene	Primer	Primer sequence (5${}^{\prime }$–3${}^{\prime }$)	GenBank accession number	Size (bp)	$T_{m}$ (${}^{\circ }$C)
*MAP3K5*	*MAP3K5*-F	TGGCCACTGGAAAACCACC	XM_042253518	264	59
	*MAP3K5*-R	GGTACTCATTCGATCCAGCTGA			
*ACTB*	*ACTB*-F	TCCGTGACATCAAGGAGAAGC	NM_001009784.3	267	52–62
	*ACTB*-R	CCGTGTTGGCGTAGAGGT			

**Figure 5:**
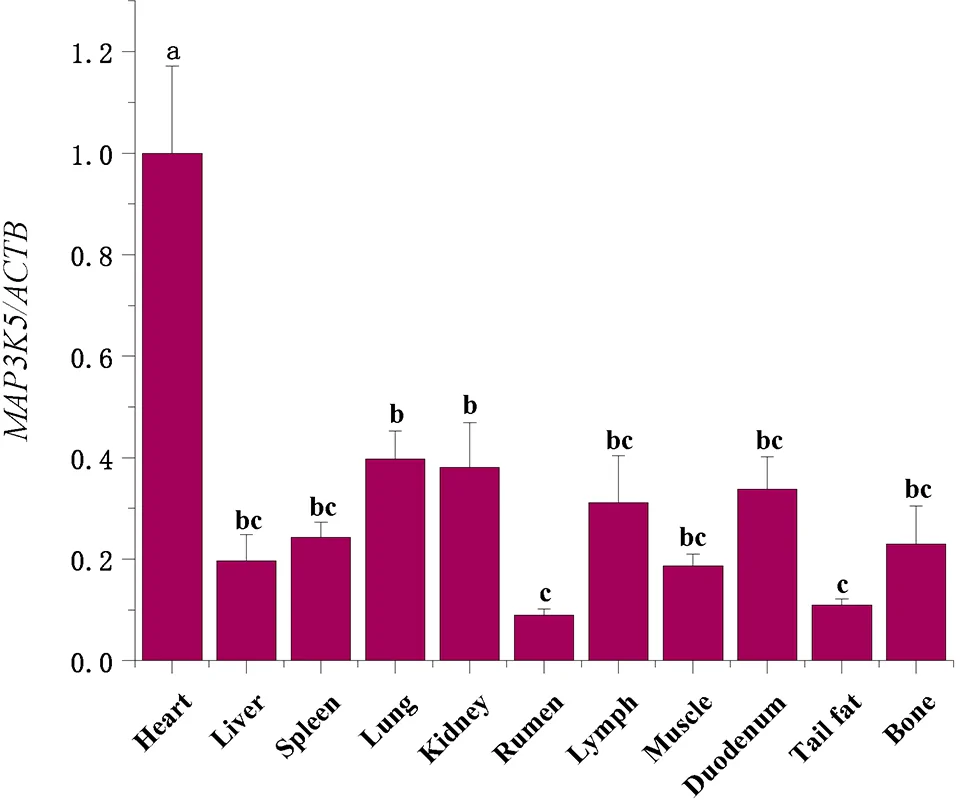
The mRNA expression profiles of the *MAP3K5* gene in various tissues of Hu sheep. Different lowercase letters indicate significant differences (P<0.05).

**Figure 6:**
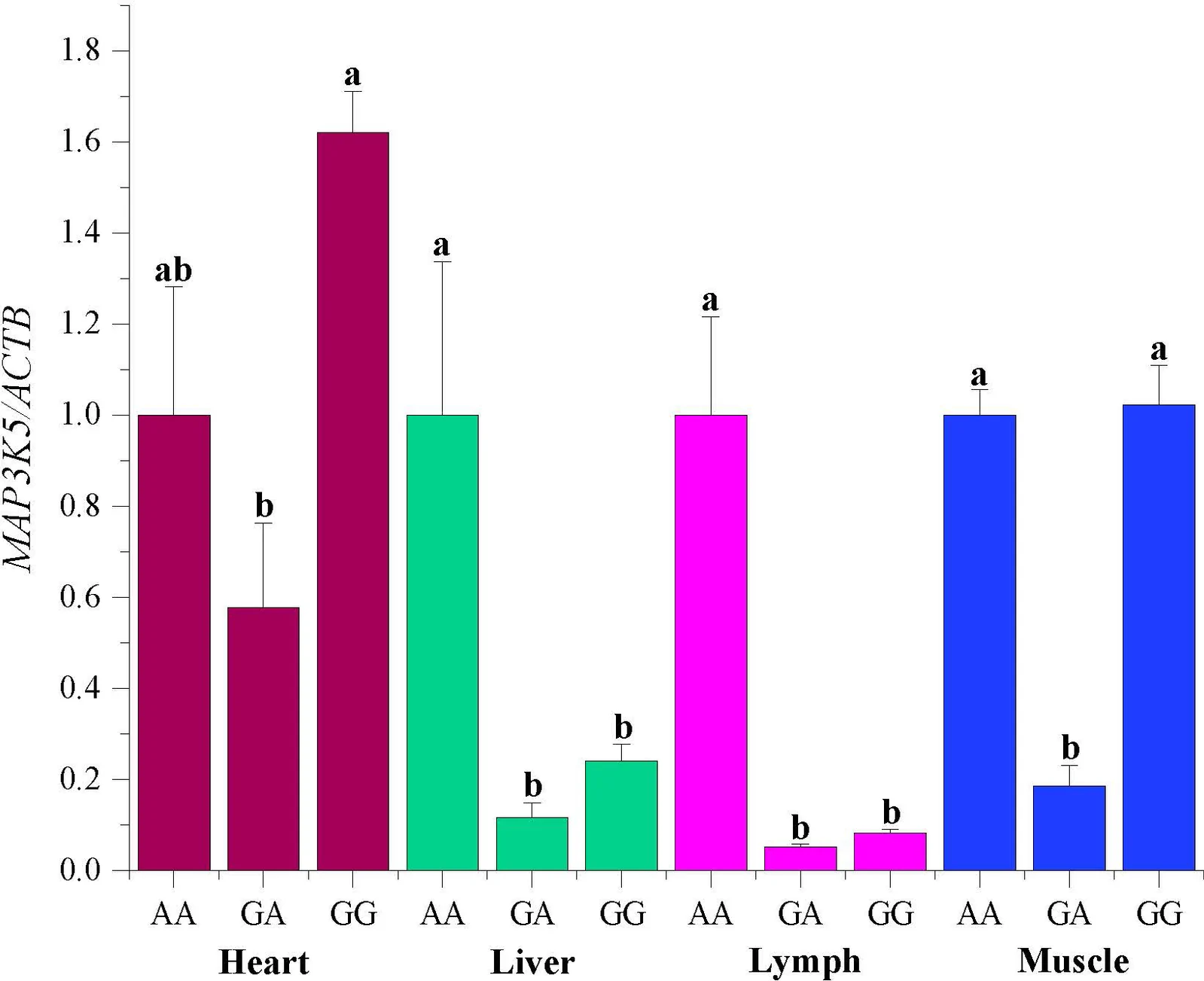
The expression level of the *MAP3K5* in different genotypes of Hu sheep. AA, GA, and GG represent Hu sheep of the AA, GA, and GG genotypes, respectively. Different lowercase letters indicate significant differences (P<0.05).

## 4 Discussion

Livestock and poultry germplasm resources and molecular breeding technology play very important roles in animal production and breeding. Therefore, it is necessary to study the association between genes and traits (Beuzen et al., 2000; Clifford et al., 2010).

This study investigated the relationship between the *MAP3K5* g.205261 A > G silencing mutation and body height, body length, chest circumference, and cannon circumference of Hu sheep. The g.205261 A > G mutation of the *MAP3K5* gene was significantly associated with the body height at 80, 100, 140, 160, and 180 d; the body length at 80 and 160 d; the chest circumference at 100, 140, 160, and 180 d; and the cannon circumference at 100 and 120 d. Similar to the results of most previous studies, the silent mutation is regarded as a kind of “silence” and does not affect the traits of the organism. However, relevant new research results show that silent mutation can cause abnormal splicing cassette stability of mRNA, thereby affecting the normal expression of protein biological functions and the activity of enzymes in the body (Nackley et al., 2006). *MAP3K5*, also known as *ASK1*, belongs to the MAPK family. *ASK1* can be activated by reactive oxygen species to produce a stress response, thereby playing an important role in cell processes such as cell survival and death (Baig et al., 2019). The study by Cho et al. (2012) found that oxidative stress is one of the most effective activators of *ASK1* (Cho et al., 2012). In this process, the MKK3/6-p38 MAPK signal performed its biological function, and finally induces the differentiation of cells. The exogenous increase of TNF-α can promote the ASK1-6-P38 MAPK signaling pathway and ultimately regulate the growth of the body. Furthermore,* ASK1* activates both JNK and p38 MAPK pathways in response to an array of stresses such as oxidative stress, endoplasmic reticulum stress, and calcium influx (Choi et al., 2011). Therefore, this cell signaling pathway is involved in the process of cell growth and differentiation in animals, thereby promoting the growth and development of animals. As a result, this gene is rarely studied in sheep, but some studies have shown that it is related to cell metabolism and cell differentiation. Therefore, it is necessary to study *MAP3K5* g.205261 A > G mutation and body size traits of Hu sheep.

According to the results of qRT-PCR, the *MAP3K5 *gene was widely expressed in these tissues. The expression level of the heart was significantly higher than those of other tissues, and there was relatively low expression in the rumen. The heart is the central organ of vertebrates, with functions such as contraction, relaxation, and pumping of blood. The role of the heart is to promote blood flow, provide sufficient blood flow to organs and tissues to supply oxygen and various nutrients, and to take away the end products of metabolism so that cells maintain normal metabolism and function. The results of RNA expression profiles were similar to the study of Pu et al. (2016) on Duroc pigs (Pu et al., 2016). At the same time, the research on mouse and chicken embryos of *ASK1* by Ferrer-Vaquer et al. (2017) also found similar results (Ferrer-Vaquer et al., 2007). Verhoeff and Mitchell (2017) found that the research on the hearts and lungs was usually combined, so it can be considered that their biological functions are also related and complementary to a certain extent (Verhoeff and Mitchell, 2017). Choi et al. (2011) found the activated *ASK1* stimulated MKK3/6–p38MAPK signaling cascade to induce specific myogenic. p38 MAPK is a core signaling molecule in myogenic differentiation (Choi et al., 2011). Chun et al. (2000) demonstrated that p38 MAPK is activated in cardiac myoblast and positively regulates myogenic genes (Chun et al., 2000). Zhang et al. (2016) found that the *MLY4* gene has the highest expression level in the hearts of small-tailed Han sheep and Dorper sheep (Zhang et al., 2016). Our results are consistent with previous research. As vital metabolic and immune organs of the body, the heart, liver, and lymph play a vital role in the healthy growth of animals. The liver is a crucial hub for many physiological processes. It is a unique metabolic and immune organ in the body that fully plays the role of oxidation, storage of liver sugar, secretory protein synthesis, trace element metabolism, detoxification, hematopoietic function recovery, and other functions (Racanelli and Rehermann, 2006; Jackson, 2017; Trefts et al., 2017). The lymph circulates through the lymphatic vessels and finally into the veins, through which part of the tissue fluid flows into the blood. Lymph is found in various parts of the animal body and plays a vital role in the body's immune system (Swartz, 2001). Therefore, we speculated that the high expression of liver and lymph can help the heart function better, so that the Hu sheep can maintain a healthy physiological state. The body's metabolism can always be at a normal level, to avoid the influence of diseases, external factors, and other factors that cause Hu sheep to be sub-healthy. Or when confronted with stimulation, the body can metabolize the toxins and other undesirable substances produced by stimulus more quickly, so that Hu sheep can maintain a better mental state (Lu et al., 2019; Orman et al., 2011; Clement et al., 2011). Therefore, the growth of AA-genotype Hu sheep is relatively better than that of GG-genotype Hu sheep. So the sheep's high expression level of the heart, liver, and lymph may be related to high metabolic capacity, highly effective immunity, high-efficiency blood circulation, and metabolism, to enable efficient utilization and processing of nutrients and metabolic wastes in the body, thereby promoting the growth of the animal's body. We speculate that the sheep *MAP3K5* gene silent mutation may influence body size traits by participating in the metabolic pathway. The *MAP3K5* gene may be used as a candidate gene that affects the body size traits of sheep. And its deep molecular mechanism of action needs further experiments to study and verify it.

## 5 Conclusions

In summary, the *MAP3K5* g.205261 A > G SNP site was identified in this study, which was related to the body height, body length, chest circumference, and cannon circumference of Hu sheep. The qRT-PCR results indicated that the expression level in the heart was the highest. Therefore, the *MAP3K5* gene may be used as a molecular genetic marker to improve the production performance of Hu sheep.

## Supplement

10.5194/aab-66-71-2023-supplement The supplement related to this article is available online at: https://doi.org/10.5194/aab-66-71-2023-supplement.

## Data Availability

The data are available from the corresponding author upon request.
