# Supplementary material for: Genetic polymorphism of the ovine MAP3K5 gene and its association with body size traits in Hu sheep of China
Source: Arch Anim Breed. 2023 Feb 16;66(1):71–9. doi: 10.5194/aab-66-71-2023 (PMC13632541; doi:10.5194/aab-66-71-2023)
Supplement: Supplementary file 1 [file aab-66-71-2023-supplement.pdf]

Supplement of Arch. Anim. Breed., 66, 71–79, 2023
https://doi.org/10.5194/aab-66-71-2023-supplement
© Author(s) 2023. CC BY 4.0 License.

Open Access
Archives Animal Breeding

Supplement of

Genetic polymorphism of the ovine *MAP3K5* gene and its association with body size traits in Hu sheep of China

Xiaobin Yang et al.

Correspondence to: Xiaoxue Zhang (zhangxx@gsau.edu.cn)

The copyright of individual parts of the supplement might differ from the article licence.

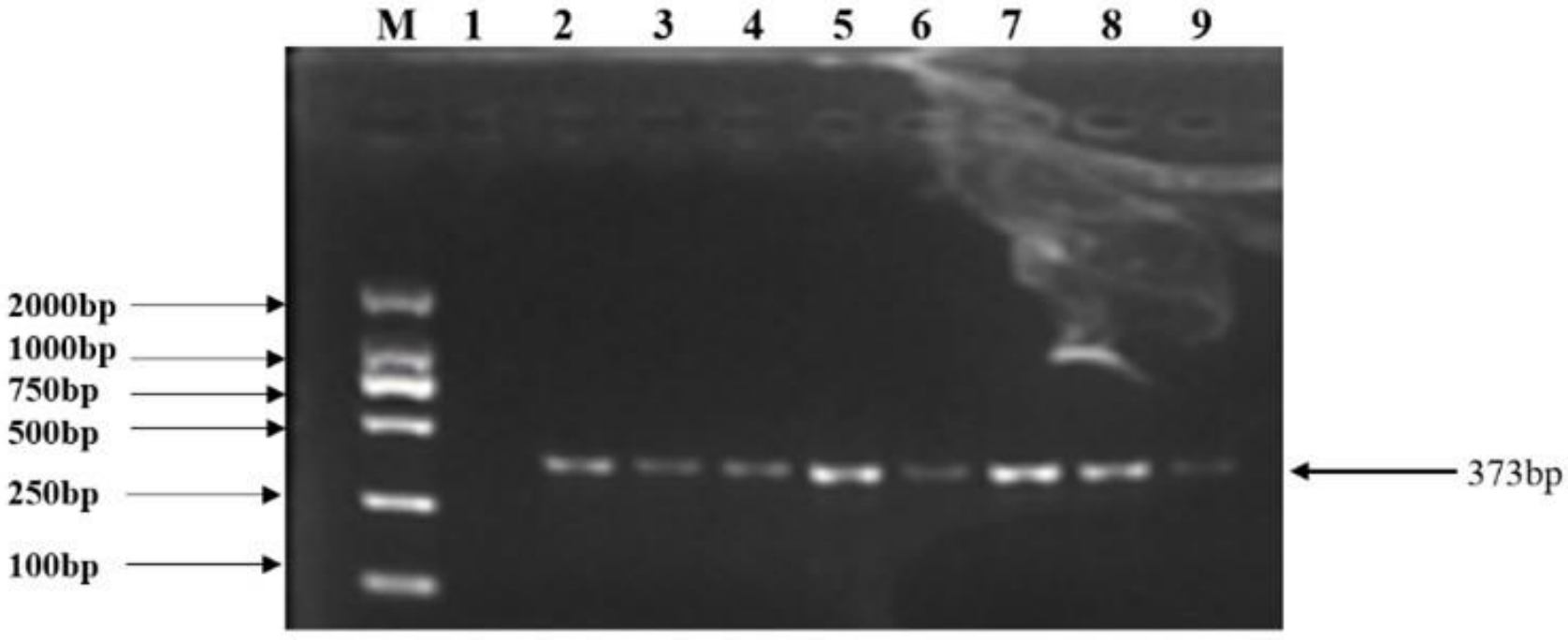

Supplementary Figure S1 a. *MAP3K5* gene optimum temperature screening PCR amplification diagram. Lanes 1-9: target fragments; M: DL2000 DNA Marker.

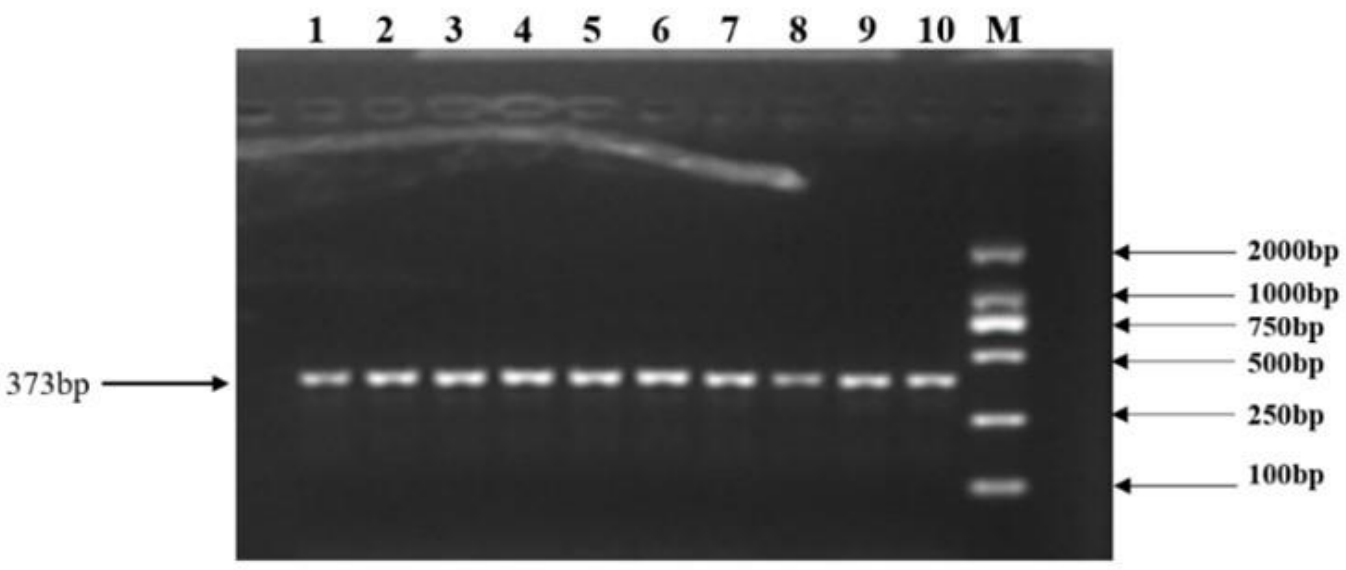

Supplementary Figure S1 b. *MAP3K5* gene optimum temperature screening PCR amplification diagram. Lanes 1-10: target fragments; M: DL2000 DNA Marker.
